# Oxidant status, iron homeostasis, and carotenoid levels of COPD patients with advanced disease and LTOT

**DOI:** 10.1080/20018525.2018.1447221

**Published:** 2018-03-09

**Authors:** M. Kentson, P. Leanderson, P. Jacobson, H. L. Persson

**Affiliations:** a Division of Medicine, Ryhov Hospital, Jönköping, Sweden; b Department of Medical and Health Sciences (IMH), Linköping University, Linköping, Sweden; c Department of Occupational and Environmental Medicine, Department of Clinical and Experimental Medicine (IKE), Linköping University, Linköping, Sweden; d Department of Respiratory Medicine, Department of Medical and Health Sciences (IMH), Linköping University, Linköping, Sweden

**Keywords:** Antioxidants, free radicals, hypoxemia, inflammation, long-term oxygen therapy

## Abstract

**Background**: The pathogenesis of chronic obstructive pulmonary disease (COPD) is associated with oxidative stress. Both iron (Fe) and oxygen are involved in the chemical reactions that lead to increased formation of reactive oxygen species. Oxidative reactions are prevented by antioxidants such as carotenoids.

**Objective**: To study the differences in Fe status, carotenoid levels, healthy eating habits, and markers of inflammation and oxidative damage on proteins in subjects with severe COPD ± long-term oxygen therapy (LTOT) and lung-healthy control subjects.

**Methods**: Sixty-six Caucasians with advanced COPD (28 with LTOT) and 47 control subjects were included. Questionnaires about general health, lifestyle, and dietary habits were answered. Lung function tests and blood sampling were performed.

**Results**: COPD subjects (±LTOT) did not demonstrate increased oxidative damage, assessed by protein carbonylation (PC), while levels of soluble transferrin receptors (sTfRs) were slightly elevated. Soluble TfRs, which is inversely related to Fe status, was negatively associated with PC. Levels of carotenoids, total and β-cryptoxanthin, α- and β-carotenes, were significantly lower in COPD subjects, and their diet contained significantly less fruits and vegetables. Lutein correlated inversely with IL-6, lycopene correlated inversely with SAT, while β-carotene was positively associated with a Mediterranean-like diet.

**Conclusions**: Fe could favor oxidative stress in COPD patients, suggesting a cautious use of Fe prescription to these patients. COPD subjects ate a less healthy diet than control subjects did and would, therefore, benefit by dietary counseling. COPD patients with hypoxemia are probably in particular need of a lycopene-enriched diet.

## Introduction

Chronic obstructive pulmonary disease (COPD), which is an inflammatory airway disease, is characterized by symptoms from the respiratory tract, particularly dyspnea in advanced stages, and a not fully reversible airflow limitation [1,2]. The impact of dietary habits, nutritional status, and nutritional interventions is increasingly emphasized due to the possible role these may play on COPD outcome and prognosis [3,4]. Several studies have evaluated the impact of nutritional therapy in COPD patients [3–5]. However, it is still unclear how specific dietary components may influence lung function [6] and important clinical outcomes in patients with COPD [7]. In focus of many studies is the possible relationship between nutritional intake, inflammation, and oxidative stress in patients with COPD [8].

Oxidative stress, defined as the imbalance between an excess of oxidants and insufficient antioxidant resources, is supposed to play a major role in COPD development [9,10]. This is because of the content of oxidants and reactive metals, mainly iron (Fe), delivered to the lung by every breath of tobacco smoke and by the inflammatory reaction that follows upon such exposure [9,10]. Reactive oxygen species (ROS) are generated by inflammatory cells in the lung in response to different stimuli, including tobacco smoke [9,10]. Thus, leucocytes from smokers release increased amounts of superoxide anions and hydrogen peroxide (H_2_O_2_) compared with leucocytes from non-smokers [9,10]. In the presence of reactive Fe, very reactive hydroxyl radicals are formed from H_2_O_2_ through Fenton reactions [9,10]. The hydroxyl radicals exert powerful oxidant injury upon surrounding tissues [11,12]. A leakage of inflammatory mediators into the capillaries of the lungs results in a systemic inflammatory response [9,10]. Thus, raised levels of systemic inflammatory markers, such as the C-reactive protein (CRP), are associated with increased all-cause mortality, cancer, and cardiovascular death [13].

Whole fruits and vegetables are rich in carotenoids. Carotenoids are antioxidants and as such associated with decreased incidence of various inflammatory disorders and chronic diseases related to oxidative stress [14]. However, these protective effects of carotenoids may not necessarily be exerted on lung tissues in the smoking human. Indeed, plenty of studies have documented potentially harmful effects of carotenoids on lung outcomes, with increased risk for lung cancer and mortality in heavy smokers [15–19].

In the present work, we have studied the relations between oxidative stress, Fe homeostasis, and levels of carotenoids in patients with advanced COPD and healthy controls. We paid a particular interest to examine patients with very low blood oxygenation saturation (SAT), thus, depending on long-term oxygen therapy (LTOT). We hypothesized that SAT measured at air breathing might be of particular importance, considering that COPD is an oxidative stress-related disease. By the present study, we wanted to shed further light on the beneficial (or not) effects of Fe-supplementation and a diet rich in carotenoids for patients with advanced COPD ± LTOT.

## Materials and methods

### Study population

Altogether, 47 lung-healthy, age- and gender-matched control subjects and 66 COPD outpatients from a single hospital (Linköping University Hospital) with stable COPD, forced expiratory volume the first second of a forced expiration (FEV_1_)/forced vital capacity (FVC) < 0.7, FEV_1_% of predicted <80% without a history of autoimmune diseases or active cancer in the past 5 yrs were included. All subjects had given written informed approval prior to inclusion. Ongoing LTOT was used to define COPD subjects with low SAT. All included COPD subjects with LTOT fulfilled the criteria for chronic respiratory failure and the Swedish guideline for prescription of LTOT, i.e. a COPD patient presenting with paO_2_ < 7.4 kPa at rest and on air breathing. All subjects used LTOT ≥ 16 h per day. The study was approved by the regional Ethical Committee (Linköping, Östergötland, Sweden; Dnr: 2012:32–31, 2012:134–32).

### Health and constitutional factors

A spirometer from Jaeger (Jaeger Vyntus Spiro, Jaeger Company, Wurzburg, Germany: using Hedenström as a reference) was used to measure lung volumes and an ABL90 Radiometer (TrioLab, Mölndal, Sweden) was used for blood gas analysis. A RAD-5 pulsoxymeter (Massimo Corp, Irvine, CA, USA) was used to measure SAT. Comorbidities, use of medications and supplements, smoking habits, and exacerbations were recorded. Comorbidities were graded using the Charlson Comorbidity Index (CCI) [20] and BMI (kg/m^2^) was calculated.

### Dietary habits

Dietary habits were assessed with a questionnaire in two different ways. First, each subject was asked to specify their average size of a typical lunch or dinner portion. This was done with four photos of plates with more or less of the three food types: (a) potatoes/rice or pasta; (b) meat or fish; and (c) vegetables. The subject then indicated what best represented their own portion on a scale from 1 (a small portion) to 4 (a very large portion). Second, the diet’s resemblance to a traditional Mediterranean diet was assessed with an 11-item questionnaire. This gave partial scores for each of the 11 individual food types and a Mediterranean Diet Score (MDS), which ranged from 0 to 44 [21].

### Laboratory measurements

Venous peripheral blood samples were drawn for immediate analysis. After centrifugation, plasma was transferred to micro tubes that were stored at −80°C. Plasma protein carbonylation (PC) was analyzed with an ELISA method that previously has been described by Buss et al. [22]. Carotenoids were analyzed with high-performance liquid chromatography and absorbance detection according to a technique described by Lidebjer et al [23].

### Statistical analysis

The results are reported as the means ± 1 SD for continuous variables. Chi-square test was used to compare nominal data, while Mann–Whitney U-test was used for ordinal data and *t*-test at normalized data on the least interval scale. Two-sided *t*-tests were used for continuous variables and Pearson’s chi-squared test for categorical variables. Correlations were analyzed by Pearson correlation coefficient.

## Results

### Differences between control subjects and COPD subjects

Forty-seven age- and gender-matched lung-healthy subjects (hereafter referred to as ‘control group/subjects’) and 66 COPD subjects completed the study. The Table 1 presents the characteristics of the study population.

**Table 1.** Characteristics of the study sample controls versus COPD subjects.

Characteristics	Control	COPD	*P*-value
Subjects, *n*	47	66	
Sex, % males	32	45	ns
Mean age, yrs, mean (SD)	70 (10)	70 (9)	ns
Never-smoker; %/*n*	58/27	8/5	<0.001
BMI (kg/m^2^); mean (SD)	25.9 (3.4)	26.3 (7.1)	ns
CCI 0–3 (%/*n*)	100/47	83/55	<0.05
CCI ≥ 4 (%/*n*)	0	17/11	<0.05
FEV_1_% predicted; mean (SD)	105 (16)	41 (16)	<0.001
SAT, % (air); mean (SD)	98 (2)	91.6 (6.0)	<0.001
LTOT; %/*n*	0/0	42/28	
Portion size, vegetables; mean (SD)	2.1 (0.6)	1.8 (0.6)	<0.05
MDS; mean (SD)	21.9 (3.9)	18.7 (3.7)	<0.0001
PC; mean (SD) (nmol/mg protein)	0.326 (0.018)	0.321 (0.006)	ns
General			
Hemoglobin; mean (SD) (ref: 134–170 g/L)	na	143 (13)	
Creatinine; mean (SD) (ref: 49–90 µmol/L)	na	78 (25)	
Albumin; mean (SD) (ref: 36–45 g/L)	na	38.1 (3.3)	
Urea; mean (SD) (ref: 3.5–8.2 mmol/L)	na	7.3 (3.1)	
Inflammation			
WBC; mean (SD) (ref: 3.5–8.8 × 10*9/L)	na	9.0 (2.6)	
Hs-CRP; mean (SD) (ref: <3 mg/L)	na	7.3 (13.3)	
Fibrinogen; mean (SD) (ref: 2–4 g/dL)	na	3.7 (1.0)	
IL-6; mean (SD) (ref: <7 ng/L)	na	6.9 (12.8)	
Fe			
sTfRs; mean (SD) (ref: 1.2–2.9 mg/L)	na	3.00 (1.14)	
Fe; mean (SD) (ref: 9–34 µmol/L)	na	14.37 (6.30)	
Transferrin; mean (SD) (ref: 1.9–3.3 g/L)	na	2.43 (0.40)	
Ferritin; mean (SD) (ref: 34–275 µg/L)	na	161.0 (153.3)	
Carotenoids, mean (SD) (µmol/L)			
Lutein;	0.48 (0.21)	0.36 (0.25)	ns
β-cryptoxanthin	0.10 (0.06)	0.07 (0.08)	<0.05
Lycopene	0.48 (0.21)	0.41 (0.20)	ns
α-carotene	0.23 (0.18)	0.09 (0.06)	<0.001
β-carotene	0.68 (0.39)	0.38 (0.21)	<0.001
Total carotenoids	1.96 (1.00)	1.31 (0.53)	<0.001

Results are presented as means (±1 S.D.) for continuous variables and percentage for categorical variables. *P*-values are from chi-square test (nominal data), Student’s *t*-test (normally distributed data), or Mann–Whitney U-test (not normally distributed data). na: not analyzed; ns: not significant (*p*-value ≥ 0.05).

BMI: body mass index; CCI: Charlson Comorbidity Index; Fe: iron; FEV_1_: forced expiratory volume in one second % predicted (according to Hedenström); hs-CRP: high-sensitivity C-reactive protein; IL-6: interleukin 6; MDS: Mediterranean Diet Score; PC: protein carbonylation; SAT: blood oxygenation saturation measured at air breathing; sTfRs: soluble transferrin receptors; WBC: white blood cell count; yrs, years.

Food portion size and MDS were significantly lower in the COPD group compared to the control group, while PC did not differ significantly. In the COPD group, 70% had advanced, stage III–IV disease and 42% were on LTOT. Compared to reference values, COPD subjects demonstrated higher levels of systemic inflammatory markers (WBC, hs-CRP, fibrinogen, and IL-6). Of all Fe indices evaluated, only soluble transferrin receptors (sTfRs) in serum deviated from reference values, and were slightly elevated. The level of total carotenoids and the levels of β-cryptoxanthin, α-carotene, and β-carotene of COPD subjects were all significantly lower in the COPD group compared to the control group.

The frequency of intake of several different vegetables and fruits was significantly lower among COPD subjects compared to the control group (*p *< 0.05). COPD subjects ate smaller portions of vegetables and fruits compared to control subjects (*p* < 0.05). No significant differences regarding the frequency of intake and amount of vegetables and fruits in the diet were noted between COPD subjects with or without LTOT.

All variables presented in the table demonstrated no significant difference when COPD subjects without LTOT were compared with those with LTOT.

### The relation between Fe or carotenoids and oxidant status, systemic inflammation, and blood oxygenation

**Figure 1.**
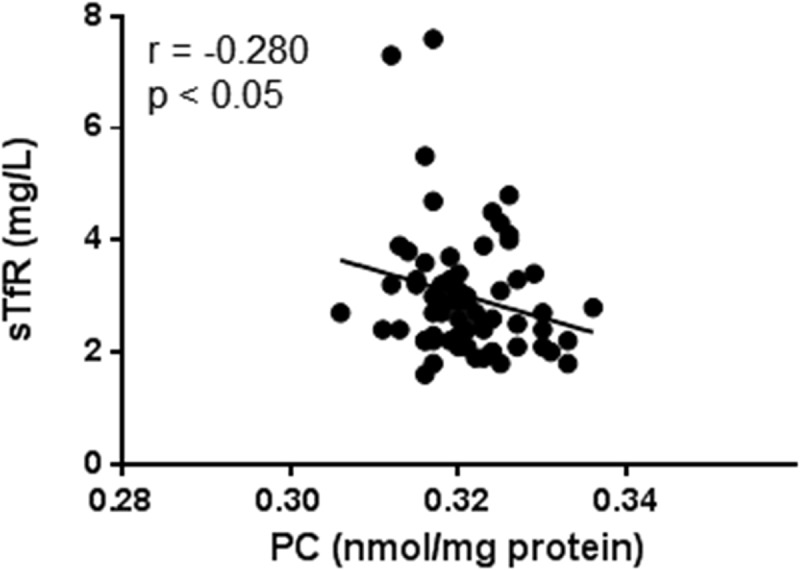
Correlation between PC and sTfRs in serum in all COPD subjects (*n* = 66). Pearson’s coefficient of correlation was calculated as indicated. sTfR: soluble transferrin receptors; PC: protein carbonylation.

The only Fe index deviating from the reference value, by being slightly elevated, was sTfRs in serum. As the level of sTfRs increases in response to lack of Fe and decreases in response to Fe excess, sTfRs is a very sensitive inverse marker of whole-body Fe deposits. Although we did not find a significant difference between COPD and control subjects regarding the level of PC in serum, notably, sTfRs was negatively associated with PC (*r* = −0.280, *p* < 0.05; Figure 1), suggesting a direct relationship between body-Fe and oxidative stress.

**Figure 2.**
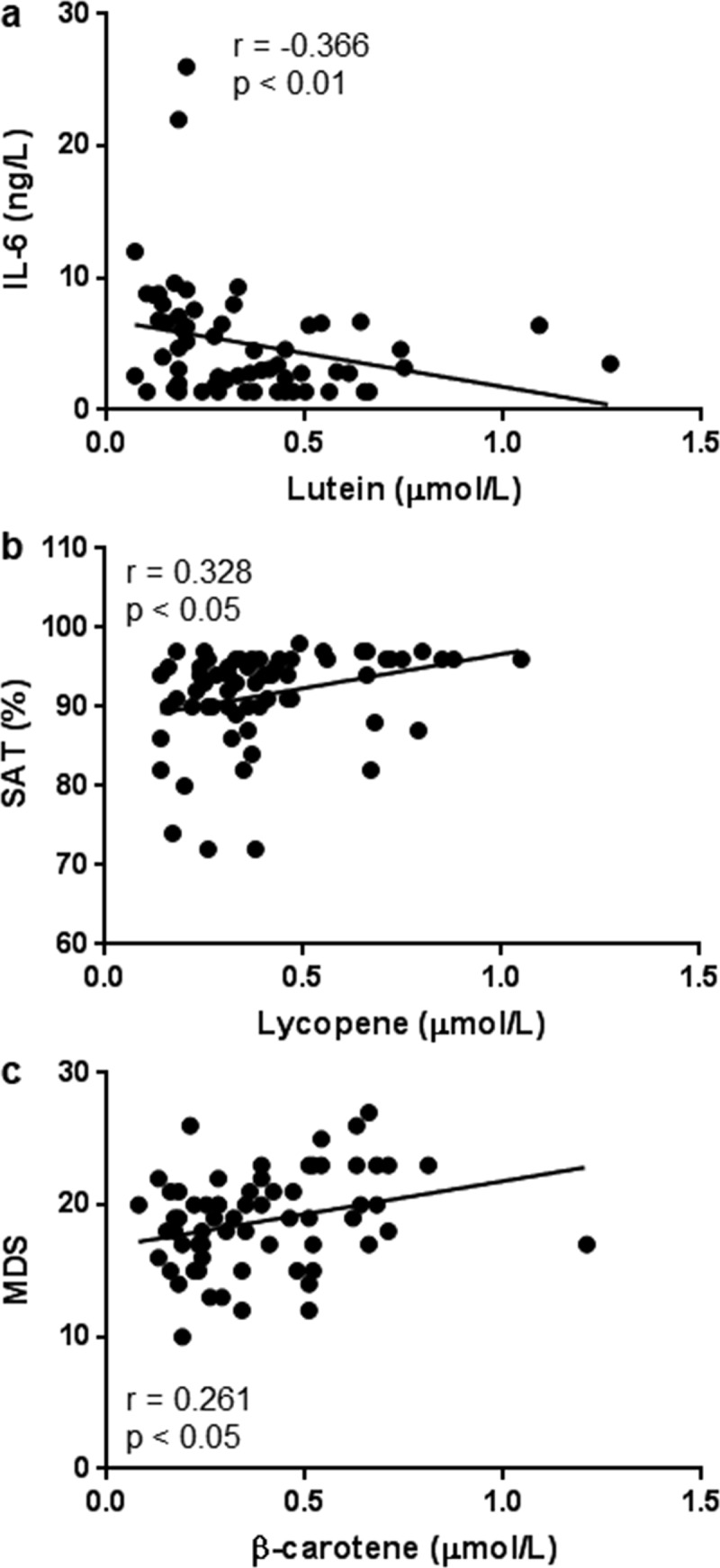
Correlations between lutein and IL-6 (a), lycopene and SAT (b), and β-carotene and MDS (c) in all COPD subjects (*n *= 66). Pearson’s coefficient of correlation was calculated as indicated. IL-6: interleukin 6; MDS: Mediterranean Diet Score; SAT: blood oxygen saturation.

Total carotenoids were positively associated with WBC (*r* = 0.286, *p* < 0.05), but negatively associated with ferritin (*r* = −0.454, *p* < 0.001). The carotenoid lutein was positively associated with WBC (*r *= 0.256, *p* < 0.05) and hs-CRP (*r* = 0.273, *p *< 0.05), but negatively associated with IL-6 (*r* = −0.366, *p* < 0.01; Figure 2(a)). The carotenoids lycopene and β-carotene were negatively associated with ferritin (*r *= −0.330, *p *< 0.05 and *r* = −0.412, *p *< 0.01, respectively).

Lycopene was positively associated with SAT (*r* = 0.328, *p* < 0.05; Figure 2(b)), while β-cryptoxanthin was negatively associated with SAT (*r* = −0.316, *p* < 0.05). Among the carotenoids levels only β-carotene positively and significantly correlated with the intake of Mediterranean-like food (*r* = 0.261, *p* < 0.05; Figure 2(c)).

## Discussion

By including and investigating COPD subjects with LTOT, the present study is the first to show a correlation between SAT and carotenoid levels in COPD subjects, i.e. a positive correlation between lycopene and SAT (Figure 2(b)). Thus, in subjects with low SAT, i.e. LTOT subjects breathing air, the level of lycopene is particularly low. A low level of lycopene may be due to hypoxemia per se or hypoxemia being a reflection of increased COPD severity. However, this is a clinically relevant observation that emphasizes the need to individualize the diet of every patient with COPD. It is therefore possible that health professionals should encourage COPD patients with ongoing LTOT to increase their intake of processed tomato products or other lycopene rich foods. Moreover, the present study indicates an anti-inflammatory effect of lutein in patients with advanced COPD. Although the plasma level of lutein did not differ significantly between COPD and control subjects, it was negatively associated with IL-6 (Figure 2(a)). It has recently been shown that lutein could act anti-inflammatory and play a role in chronic disorders such as coronary artery disease [24]. In that study, the plasma level of lutein was inversely associated with IL-6 in serum from patients with coronary artery disease and lutein did also attenuate the release of inflammatory cytokines by lipopolysaccharide-treated peripheral blood mononuclear cells *ex vivo*.

Evidence of systemic oxidative damage, previously reported in smokers and COPD patients, is based on observations done by a variety of methods such as assessment of activity of glutathione peroxidase in erythrocytes, levels of oxidative DNA damage in blood leucocytes (e.g. 8-hydroxydeoxyguanosine), lipid peroxidation products, and nitrated proteins in plasma [25,26]. In the present study, we used PC in plasma as an index for systemic oxidative status, but could, however, not find any significant difference between controls and COPD subjects. Other research groups have shown a significant difference using PC in plasma, but these studies were larger than the present study [27,28].

In the present study, we used sTfRs in serum to estimate whole-body Fe deposits. Indeed, the use of sTfRs improves the clinical diagnosis of Fe deficiency in the presence of a coexisting chronic inflammatory disease such as COPD [29]. The concentration of sTfRs is an indicator of Fe status that relates inversely to the Fe deposits [29]. Thus, Fe deficiency will result in an increase in sTfRs levels, while Fe repletion will cause a decrease in sTfRs levels [29]. While ferritin is the major protein for storage and, consequently, increases in serum in response to Fe excess, ferritin is also an acute-phase reactant and elevates in response to processes that do not correlate with Fe status, such as inflammation [29]. In contrast, sTfRs is not an acute-phase reactant; thus, the interpretation of the level of sTfRs in serum depends solely on the present Fe status [29]. Thus, applying this method to define Fe status, we found that sTfRs correlated negatively with PC, indicating that PC would correlate positively with whole-body Fe deposits (Figure 1). One could, therefore, hypothesize that increased deposits of body Fe could elevate an oxidant burden and that the following oxidative stress may then worsen the disease further. Today, most pulmonary physicians cautiously prescribe Fe to patients with advanced COPD and concomitant hypoxemia. This is because of the risk for these individuals to develop erythrocytosis secondary to the elevated erythropoietin levels present. However, the results presented here indicate that Fe-driven oxidant damage might be another matter of concern in these patients and that this also has to be considered before Fe supplementation.

In previous studies, cigarette smoking has been associated with decreased plasma β-carotene level [30–32]. It has also been shown that β-carotene treatment may accelerate the development of lung cancer in cigarette smokers [33,34]. In line with previous studies, the present study demonstrates significantly decreased β-carotene plasma levels among COPD subjects compared to the control group. However, β-carotene was positively associated with the intake of Mediterranean-like food (Figure 2(c)), suggesting a positive role for β-carotene in COPD. A Mediterranean diet is considered healthy because of its low proportion of saturated fat, red meat, and refined grains and a high proportion of fruits, vegetables, whole grains, legumes, nuts, monounsaturated fat, and fish. Mediterranean-like food is also known to protect against all-cause mortality, coronary heart disease and diabetes [35].

In our study, subjects in the COPD group had a significantly lower intake of fruit and vegetables. Previously, an inverse association between consumption of fruits, but not vegetables, and COPD mortality was observed [36]. Conversely, a diet characterized by high consumption of fruits, vegetables, whole grains, polyunsaturated fatty acids, nuts, and long-chain omega-3 fats and low consumption of red and processed meat, refined grains, and sugar-sweetened drinks was associated with a lower risk of COPD in men and women in the United States [37]. Moreover, data from a large population-based prospective cohort of men showed that high consumption of fruits and vegetables is associated with reduced COPD incidence in both current and ex-smokers but not in never-smokers [38]. Thus, most data today support the idea that a high daily intake of fruits and vegetables is favorable for patients with advanced COPD.

A major limitation of the present study needs to be addressed. Instead of relying on a single method (PC) for assessment of oxidant damage in serum, we should have used a second or even a third method for this purpose. In contrast, systemic inflammation was assessed using several variables in blood and serum, i.e. WBC, fibrinogen, hs-CRP, IL-6, and ferritin, the latter also being a Fe indices. Fibrinogen did not significantly correlate with any of the variables used to reflect oxidant status, Fe homeostasis and carotenoids. WBC was less useful, due to the influence of ongoing steroid therapy in most COPD subjects, which elevates the number of circulating leucocytes in the blood. Hs-CRP and ferritin, both being acute-phase reactants, respond to inflammation regardless of the disease behind the systemic inflammation. This possibility was accounted for by excluding study subjects with known autoimmune disease and ongoing immune-modulating therapy (except steroids). Collectively, the relationships between the levels of IL-6 and ferritin and the levels of carotenoids suggest an anti-inflammatory role of carotenoids. However, the level of lutein correlated both ways to inflammatory markers, negatively to IL-6, and positively to hs-CRP. In COPD subjects, the level of hs-CRP may reflect disease severity better than the level of IL-6, as was suggested by a recent meta-analysis study [39]. In that study, serum levels of CRP correlated negatively and significantly to FEV_1_ in COPD subjects (*r *= −0.13, *p *= 0.012), while serum levels of IL-6 did not [39].

## Conclusion

Fe could favor oxidative stress in COPD patients, suggesting a cautious use of Fe prescription to these patients. COPD subjects ate a less healthy diet than control subjects did and would, therefore, benefit by dietary counseling. COPD patients with hypoxemia are probably in particular need of a lycopene-enriched diet.
